# Lipoblastome découvert chez le grand enfant: description rare et revue de la littérature

**DOI:** 10.11604/pamj.2017.28.119.12002

**Published:** 2017-10-06

**Authors:** Abdoulaye Diallo Harouna, Franck Mvumbi, Karima Atarraf, Lamiae Chater, Meryem Boubbou, Abderrhmane Afifi

**Affiliations:** 1 Service de Traumato-orthopédie Pédiatrique, CHU-Hassan II, Fès, Maroc; 2 Service de Radiologie Mère et Enfant, CHU-Hassan II, Fès, Maroc; 3 Université Sidi Mohamed Ben Abdellah, Faculté de Médecine et de Pharmacie de Fès, Maroc

**Keywords:** Lipoblastome, tumeur adipeuse, grand enfant, Lipoblastoma, badipose tumor, large child

## Abstract

Le lipoblastome est une tumeur bénigne relativement rare. Il est issu de la graisse blanche embryonnaire qui survient presque exclusivement chez le petit enfant, de moins de 3 ans. Nous rapportons un cas de lipoblastome de la cuisse gauche découvert chez un grand enfant (11 ans), avec une revue de la littérature. Le diagnostic était histologique, et le traitement chirurgical consistait à une exérèse totale de la masse. Les suites post-opératoires étaient simples avec un recul de 9 mois.

## Abstract (en)

Lipoblastoma is a relatively rare benign tumor. It derives from embryonic white fat cells. It almost exclusively affects children less than 3 years of age. We report a case of lipoblastoma of the left thigh detected in an older child (11 years) and a literature review. Diagnosis was based on histology while surgical treatment was based on total resection of the mass. The postoperative course was simple with a follow-up period of 9 months.

## Introduction

Le lipoblastome est une tumeur bénigne rare de la graisse blanche d’origine embryonnaire. Il apparait presque exclusivement chez les petits enfants (de moins de 3 ans) [1]. Il existe une légère prédominance masculine. A la date d’aujourd’hui, moins de 200 cas ont été rapportés à travers la littérature [2]. Elle peut se présenter soit comme une tumeur bien délimitée, encapsulée (lipoblastome bénin), soit non encapsulée, infiltrante et diffuse (lipoblastomatose) [1, 2].

## Patient et observation

**Figure 1:**
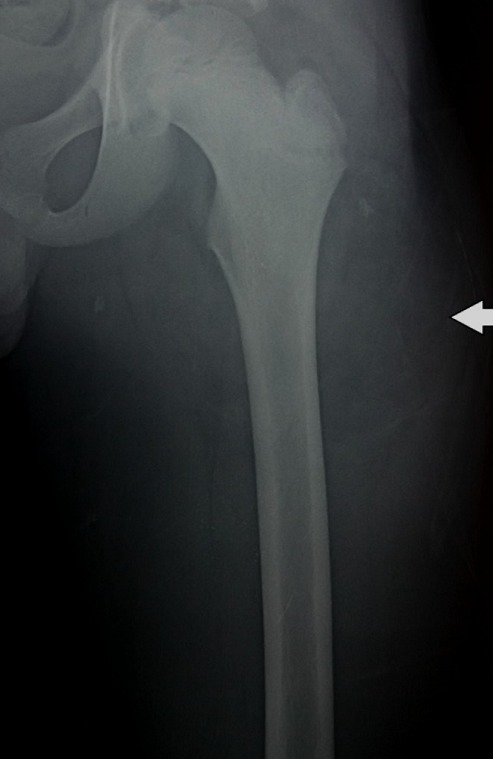
Radiographie standard de la cuisse gauche montrant une opacité des parties molles latérales externes sans anomalie osseuse en regard

**Figure 2:**
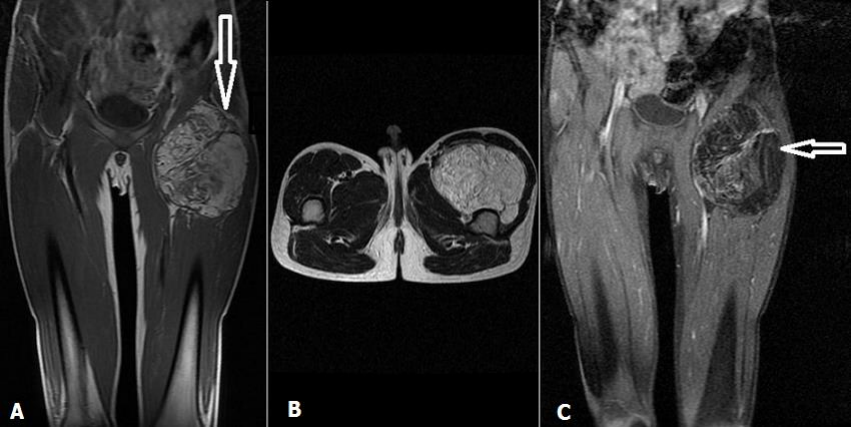
IRM: séquences pondérées en T1 sans (figure A et B) et avec FATSAT injecté (C), séquence pondérée T2 objectivant une volumineuse masse de la cuisse gauche hyper-signal T1 et T2 hétérogène rehaussée faiblement après contraste et s’effaçant après saturation de la graisse

**Figure 3:**
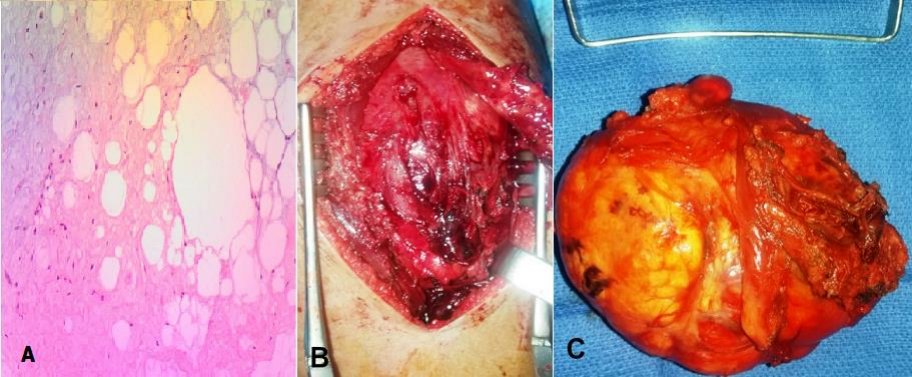
Biopsie préopératoire objectivant une prolifération adipeuse constituée par des adipocytes réguliers, disposés en nodules de tailles variables dont les noyaux sont peu visibles; on retrouve également des nombreuses cellules étoilées de petite taille. Ces dernières se disposent sur un fond myxoïde entre les adipocytes: A) elles ne présentent pas de signes de malignité; B) vue peropératoire montrant une masse bien encapsulée refoulant les différents plans musculaires; C) volumineuse masse adipeuse bien encapsulée

Enfant âgé de 11 ans, de sexe masculin emmenait en consultation pour une tuméfaction de la cuisse gauche d’évolution progressive depuis 7 mois. Il n’avait pas de notion de fièvre ni de boiterie, l’état général était conservé. L’examen clinique retrouvait au niveau de la face antérieure du 1/3 proximal de la cuisse gauche une masse mesurant 10 cm de grand axe. Elle était de consistance ferme, indolore, sans signes inflammatoires associés, mobile par rapport au plan superficiel et fixe par rapport au plan profond. Ailleurs l’examen somatique était normal. Le bilan biologique était normal. La radiographie de la cuisse en incidence de face (Figure 1) avait montré une infiltration des parties molles sans lésions osseuses. L’étude échographique de la masse révélait la présence d’une formation bien limitée, hyper-échogène hétérogène, traversée par des septas épais et hyper-vascularisés au Doppler couleur. L’imagerie par résonnance magnétique (IRM) objectivait un processus tumoral sous aponévrotique intermusculaire de signal hétérogène avec une composante graisseuse en hyper-signal T1 (Figure 2 A et B) et T2 (Figure 2 C) s’effaçant après saturation de la graisse et une composante tissulaire en hypo-signal T1, hyper-signal T2 intermédiaire se rehaussant après injection de Gadolinium, sans lyse corticale ni anomalie de signal osseux associées. La biopsie pré opératoire de la masse était revenue en faveur d’un lipoblastome sans signes de malignité (Figure 3 A). L’indication d’une chirurgie d’exérèse tumorale a été retenue. La voie d’abord était une incision longitudinale prolongeant l’ancienne incision de la biopsie sur la face antérieure de la cuisse. L’exploration chirurgicale retrouvait une masse encapsulée très adhérente aux différents plans musculo-aponévrotiques qu’elle refoulait sans les envahir (Figure 3 B). Elle était également adhérente au périoste fémoral. La masse était reséquée complètement sans ouverture capsulaire, en suivant les plans de clivage entre celle-ci et les plans musculaires et en emportant une partie du périoste. Elle mesurait 12cm de grand axe renfermant plusieurs lobules d’aspects graisseux regroupés en amas (Figure 3 C). L’analyse histologique de la pièce d’exérèse a confirmé les résultats de la biopsie en montrant une prolifération tumorale adipeuse, constituée par des adipocytes réguliers, de grande taille dont les noyaux sont peu visibles en faveur d’un lipoblastome sans signes de malignité. Les suites opératoires étaient simples, avec un recul de 9 mois, le patient n’avait aucune plainte particulière ni de récidive locale.

## Discussion

Le lipoblastome est une tumeur adipeuse bénigne d’origine embryonnaire [1]. Il est relativement rare, à l’heure actuelle moins de 200 cas ont été rapportés à travers la littérature [1]. Elle survient quasi exclusivement chez le petit enfant de moins de 3 ans avec un âge moyen de 12 mois [1, 2]. Cette tumeur apparait rarement après l’âge de 10 ans, nous n’avons retrouvé qu’une seule description chez un enfant de 12 ans qui avait une localisation retro-péritonéal [1, 3]. Notre cas apparait comme l’une des rares cas connu de la littérature survenu après l’âge de 11 ans. Cliniquement le lipoblastome se présente comme une tumeur de taille et de croissance variables, le plus souvent de petite taille et asymptomatique. Elle peut devenir symptomatique dans certaines localisations par compression des structures avoisinantes: dyspnée, hypoxie, stridor (localisation thoracique) et œdèmes des membres inférieurs (localisation retro-péritonéale) [1]. La localisation au niveau des extrémités est de loin la plus fréquente [1, 4, 5]. Cette tumeur se présente sous deux formes soit une tumeur bien limitée, encapsulée (lipoblastome bénin), soit non encapsulée, infiltrante et diffuse (lipoblastomatose) [1–3, 6–8]. Chez notre patient, la tumeur était de localisation profonde, bien limitée et encapsulée, ce qui constitue également une description rare. Dans la plupart des cas le diagnostic préopératoire n’est pas possible. Le bilan biologique n’est pas de grand apport. La radiographie standard ne montre pas de calcifications ni de lyse osseuse. L’échographie peut révéler une masse d’écho-structure hétérogène, lobulée pouvant contenir des formations kystiques en son sein. La tomodensitométrie (TDM) ou mieux l’imagerie par résonnance magnétique (IRM) permettent de préciser la localisation, la taille, les rapports ainsi que le caractère lobulée et lipomateux [1, 4]. Tous ces caractères sus-décrits ont été retrouvés chez notre patient. Le diagnostic définitif est histopathologique à travers une biopsie chirurgicale, permettant d’éliminer une tumeur maligne par la même occasion. Le traitement est chirurgical avec exérèse totale de la lésion. L’exérèse incomplète est une source potentielle de récidive tumorale. Les suites opératoires sont généralement simples. Le pronostic est bon.

## Conclusion

Le lipoblastome est une tumeur bénigne rare, bien encapsulée presque exclusivement décrit chez les enfants de moins de 3 ans. La clinique est non-spécifique. L’imagerie par résonnance magnétique permet d’orienter le diagnostic, qui ne peut être confirmé que par l’examen histopathologique. Le traitement est chirurgical avec exérèse totale. Le pronostic est bon. A travers cet article, nous avons rapporté le cas d’un lipoblastome atypique de la cuisse gauche, survenu chez un grand enfant (âgé de plus de 11 ans) et de localisation profonde.

## Conflits d’intérêts

Les auteurs ne déclarent aucun conflit d'intérêts.

## References

[1] Harrer J, Hammon G, Wagner T, Bolkenius M (2001). Lipoblastoma and Lipoblastomatosis: a report of two cases and review of the literature. Eur J Pediatr Surg..

[2] Miller GG, Yanchar NL, Magee JF, Blair GK (1998). Lipoblastoma and liposarcoma in children: an analysis of 9 cases and a review of the literature. Can J Surg..

[3] Jimenez JF (1986). Lipoblastoma in infancy and childhood. J Surg Oncol..

[4] Yada K, Ishibashi H, Mori H, Shimada M (2016). Intrascrotal lipoblastoma: report of a case and the review of literature. Surg Case Rep..

[5] Beebe MM, Smith MD (1993). Omental lipoblastoma. J Pediatr Surg..

[6] Crozier F, Jouve JL, Zattara-Cannoni H, Bouvier C, Jaoua S, Charrier A (2002). Lipoblastoma of the buttock. J Radiol..

[7] Stock N (2015). Tumeurs adipeuses. Ann Pathol..

[8] Papillard-Maréchal S, Brisse HJ, Pannier S, Ilharreborde B, Philippe-Chomette P, Irtan S (2015). Masses des tissus mous d’allure tumorale de l’enfant et de l’adolescent. Arch Pediatr..

